# Endothelium‐specific CYP2J2 overexpression attenuates age‐related insulin resistance

**DOI:** 10.1111/acel.12718

**Published:** 2018-01-10

**Authors:** Yan Yang, Ruolan Dong, Zhihui Chen, Danli Hu, Menglu Fu, Ying Tang, Dao Wen Wang, Xizhen Xu, Ling Tu

**Affiliations:** ^1^ Department of Geriatric Medicine Tongji Hospital Tongji Medical College Huazhong University of Science and Technology Wuhan China; ^2^ Hubei Key laboratory of Genetics and Molecular Mechanisms of Cardiological Disorders and Division of Cardiology Department of Internal Medicine Tongji Hospital Tongji Medical College Huazhong University of Science and Technology Wuhan China

**Keywords:** adipose tissue, aging, CYP2J2, inflammation, insulin resistance

## Abstract

Ample evidences demonstrate that cytochrome P450 epoxygenase‐derived epoxyeicosatrienoic acids (EETs) exert diverse biological activities, which include potent vasodilatory, anti‐inflammatory, and cardiovascular protective effects. In this study, we investigated the effects of endothelium‐specific CYP2J2 overexpression on age‐related insulin resistance and metabolic dysfunction. Endothelium‐specific targeting of the human CYP epoxygenase, CYP2J2, transgenic mice (Tie2‐CYP2J2‐Tr mice) was utilized. The effects of endothelium‐specific CYP2J2 overexpression on aging‐associated obesity, inflammation, and peripheral insulin resistance were evaluated by assessing metabolic parameters in young (3 months old) and aged (16 months old) adult male Tie2‐CYP2J2‐Tr mice. Decreased insulin sensitivity and attenuated insulin signaling in aged skeletal muscle, adipose tissue, and liver were observed in aged adult male mice, and moreover, these effects were partly inhibited in 16‐month‐old CYP2J2‐Tr mice. In addition, CYP2J2 overexpression‐mediated insulin sensitization in aged mice was associated with the amelioration of inflammatory state. Notably, the aging‐associated increases in fat mass and adipocyte size were only observed in 16‐month‐old wild‐type mice, and CYP2J2 overexpression markedly prevented the increase in fat mass and adipocyte size in aged Tie2‐CYP2J2‐Tr mice, which was associated with increased energy expenditure and decreased lipogenic genes expression. Furthermore, these antiaging phenotypes of Tie2‐CYP2J2‐Tr mice were also associated with increased muscle blood flow, enhanced active‐phase locomotor activity, and improved mitochondrial dysfunction in skeletal muscle. Collectively, our findings indicated that endothelium‐specific CYP2J2 overexpression alleviated age‐related insulin resistance and metabolic dysfunction, which highlighted CYP epoxygenase‐EET system as a potential target for combating aging‐related metabolic disorders.

## 1 INTRODUCTION

Aging is a complex biological process characterized by a progressive deterioration in physiological functions and metabolic processes that increase vulnerability to death. Metabolic syndrome, an aging‐related disorder, includes a set of clinical conditions such as dyslipidemia, hyperglycemia, insulin resistance, and hypertension (Barbieri, Rizzo, Manzella & Paolisso, 2001; Ford, Giles & Dietz, 2002). Insulin resistance is a key component of the metabolic syndrome and a prelude to type 2 diabetes mellitus (T2DM). Evidence suggests that insulin sensitivity gradually decreased during the aging process in humans and animals (Catalano, Bergman & Ader, 2005; Petersen et al., 2003). However, those factors which contribute to aging‐related insulin resistance and metabolic dysfunction remain poorly understood.

Aging is accompanied by deleterious physiological changes, including reduced energy expenditure and changes in body composition favoring increased adiposity (Akasaki et al., 2014; Camporez et al., 2016). Adipose tissue mass is maintained by the balance between lipid synthesis and catabolism. Disruption in this equilibrium is implicated in the pathophysiology of various metabolic disorders, particularly obesity and type two diabetes (Guilherme, Virbasius, Puri & Czech, 2008). Furthermore, adipose tissue is associated with a chronic, low‐grade inflammation that contributes to defects in the critical nodes of insulin signaling (Palmer & Kirkland,^2016^; Tchkonia et al., 2010). In addition, aging is also associated with reductions in skeletal muscle mitochondrial function. These aging‐related reductions in mitochondrial ATP production rates (MAPRs) have also been associated with concomitant reductions in skeletal muscle mitochondrial enzyme activities and protein expression in humans and rodents (Karakelides, Irving, Short, O'Brien & Nair, 2010; Kim, Wei & Sowers, 2008; Petersen et al., 2003). However, it should be recognized that the association between insulin resistance and mitochondrial dysfunction is not consistent.

Cytochrome P450 2J2 (CYP2J2), a human epoxygenase, metabolizes arachidonic acid to four regioisomeric epoxyeicosatrienoic acids (5,6‐, 8,9‐, 11,12‐, and 14,15‐EET). EETs are expressed in livers, cardiac myocytes, vascular endothelium, pancreas, and other tissues and likely play important roles in regulating cardiovascular functions and malignancy under physiological and/or pathological conditions (Xu, Zhang & Wang, 2011). In vivo experiments have demonstrated that EETs reduce insulin resistance and metabolic syndrome in fructose‐treated rats and ob/ob and db/db mice (Burgess, Vanella, Bellner, Schwartzman & Abraham, 2012; Xu et al., 2010). Another study shows CYP2J2 overexpression attenuates the diabetic phenotype and insulin resistance by reducing hepatic inflammation via the PPARγ (Li et al., 2015). In addition, endothelium‐specific CYP2J2 overexpression alleviates high‐fat diet‐induced hyperlipidemia via increased fatty acid oxidation mediated by the AMPK pathway (Zhang et al., 2015). It has also been reported that EET‐A (an EET analogue) increased mitochondrial biogenesis and insulin sensitivity, thereby providing metabolic syndrome protection in high‐fat diet‐induced obesity in mice (Singh, Bellner et al., 2016; Singh, Schragenheim et al., 2016). These studies reinforce the importance of the development of EETs as promising drugs for the treatment of insulin resistance. Despite advances in our understanding of metabolic regulation by the EETs and the regulatory mechanisms of cytochrome P450‐mediated eicosanoids are achieved (Theken et al., 2012), little is known regarding the involvement of EETs in aging‐related metabolic dysfunction.

In this study, the effects of endothelium‐specific CYP2J2 overexpression on the metabolic dysfunction in 16‐month‐old male mice were investigated. Interestingly, our findings revealed that CYP2J2 overexpression significantly ameliorated age‐related metabolic dysfunction.

## 2 RESULTS

### 2.1 Identification of Tie2‐CYP2J2‐Tr mice

Tie2‐CYP2J2‐Tr mice on a pure C57BL/6J genetic background were obtained from Dr. Darryl Zeldin's colony. As expected, abundant CYP2J2 protein expression determined by Western blots was observed in livers, skeletal muscles, and epididymal fat of Tie2‐CYP2J2‐Tr mice (Figure S1a). However, there was no CYP2J2 expression in these tissues in their littermate control mice. In addition, serum 14, 15‐EET level in Tie2‐CYP2J2‐Tr mice was higher than that in WT mice. Interestingly, aging significantly reduced 14, 15‐EET level in serum compared with young WT controls (Figure S1b). These results indicate that Tie2‐CYP2J2‐Tr mice were successfully introduced into our laboratory and can be used in this study.

### 2.2 Tie2‐CYP2J2‐Tr mice are protected against fat accumulation and peripheral insulin resistance during aging

**Figure 1:**
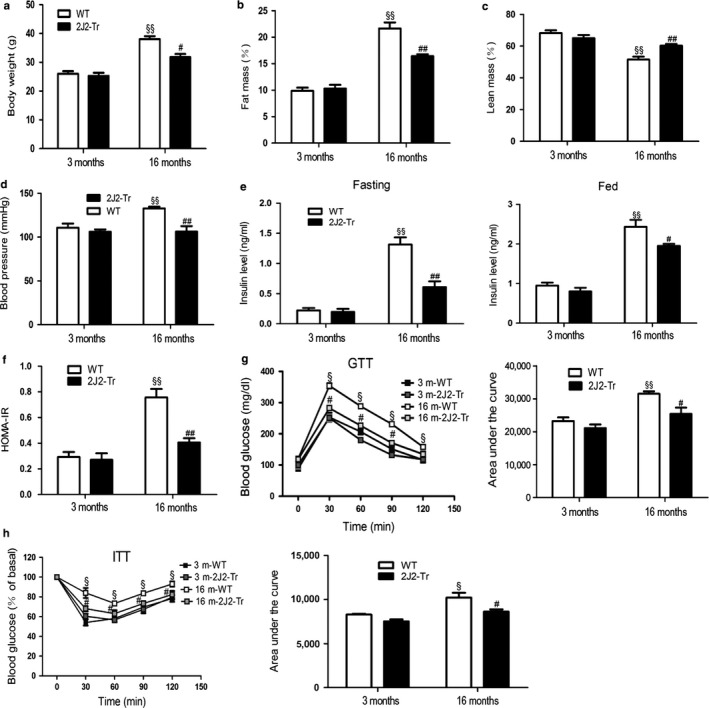
CYP2J2 overexpression protects against fat accumulation and insulin resistant during aging. (a) Total body weight of male 3‐ and 16‐month‐old wild‐type (WT) and Tie2‐CYP2J2‐Tr (2J2‐Tr) mice. (*n* = 8–10 mice in each group). (b, c) fat mass and lean mass were analyzed by Bruker's minispec LF 50 Whole Body Composition Analyzer. (d) Systolic blood pressure of the mice. (e) Plasma insulin concentrations during fasting and fed conditions. (f) Insulin resistance as measured by HOMA‐IR. HOMA‐IR index (homeostatic model assessment index of insulin resistance) was calculated as follows: HOMA‐IR index = (fasting blood glucose, mmol/L) × (fasting insulin, mU/l)/22.5. (g) GTT and corresponding AUC analysis. (h) ITT and corresponding AUC analysis. Data are shown as means ± *SE* (*n* = 8 per group). ^§^ *p *< .05,16 mWT vs. 3 mWT; ^§§^ *p *< .01, 16 mWT vs. 3 mWT; ^#^ *p *< .05, 16 m2J2‐Tr vs. 16 mWT; ^##^ *p *< .01, 16 m2J2‐Tr vs. 16mWT

Fat tissue is at the nexus of mechanisms involved in longevity and aging‐related metabolic dysfunction. Fat distribution and function change dramatically throughout life (Barbieri et al., 2001; Tchkonia et al., 2010). Considering Tie2‐CYP2J2‐Tr‐HF mice had a reduction in body weight gain and insulin levels (Abraham et al., 2014), the potential effects of endothelium‐specific CYP2J2 overexpression on aging‐related adiposity and insulin resistance were explored in this study. As shown in Figure 6a, no difference in the food intake of the four groups of mice suggests no change in energy intake. Male wild‐type mice at 16 months of age (hereafter referred to as 16‐month‐old WT mice) displayed significant increases in total body weight, percentage of fat mass, percentage of epididymal fat, and percentage of subcutaneous fat as compared with those at 3 months of age (hereafter referred to as 3‐month‐old WT mice) (Figures 1a,b and S2). Furthermore, lean mass relative to body weight in 16‐month‐old WT mice was lower than that in 3‐month‐old wild‐type mice as shown in Figure 1c. Importantly, these alterations in 16‐month‐old WT mice were partially prevented in age‐matched Tie2‐CYP2J2‐Tr mice (hereafter referred to as 16‐month‐old CYP2J2‐Tr mice). In addition, systolic blood pressure was significantly lower in 16‐month‐old CYP2J2‐Tr mice compared with that of 16‐month‐old WT mice (Figure 1d). Collectively, these data indicated that endothelium‐specific targeting of CYP2J2 markedly inhibited aging‐related adiposity.

**Figure 2:**
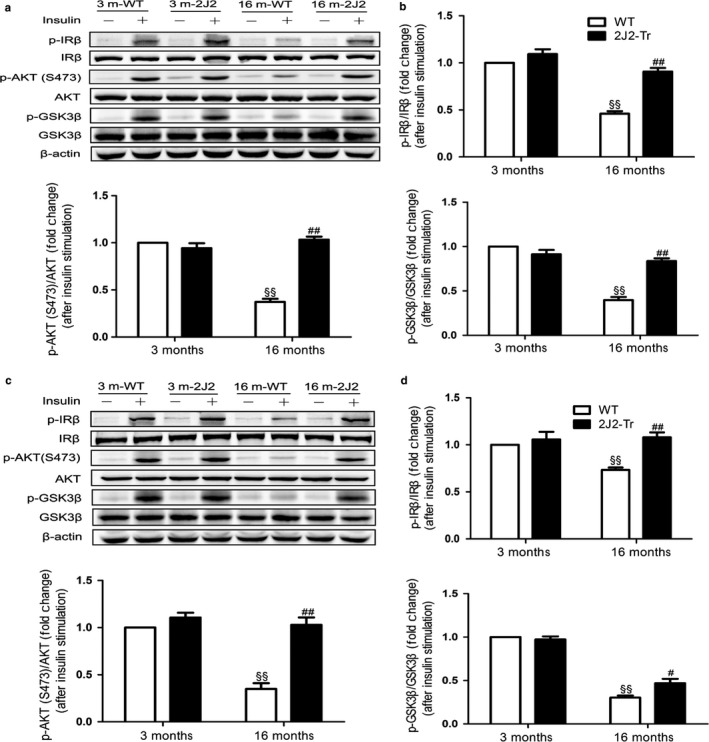
CYP2J2 overexpression recovers insulin signaling in adipose and muscle from 16‐month‐old mice. After overnight fasting, mice were anesthetized and 1 IU per kg insulin or an equal volume of vehicle was administered through the portal vein. Adipose tissue and gastrocnemius muscle were collected 120 s after the injection and analyzed by Western blot. (a, b) Representative immunoblots and quantitation of insulin signaling. Immunoblots of p‐IRβ, IRβ, p‐Akt (S473), T‐Akt, p‐GSK3β,GSK3β, and β‐actin in epididymal adipose tissue of mice at 3 or 16 month of age. (c, d) Representative blots and quantitation of insulin signaling. Immunoblots of p‐IRβ, IRβ, p‐Akt (S473), T‐Akt, p‐GSK3β,GSK3β, and β‐actin in gastrocnemius muscle of the different experimental groups of mice. Data are shown as means ± *SE* (*n* = 5–6 per group). ^§§^ *p *< .01, 16 mWT vs. 3 mWT; ^#^ *p *< .05, 16 m2J2‐Tr vs. 16 mWT; ^##^ *p *< .01, 16 m2J2‐Tr vs. 16 mWT

Consistent with this, the aging‐associated increases in fasting serum insulin level, fed insulin level, and HOMA‐IR index (a quantitative analysis to measure insulin resistance) were also partially inhibited in the age‐matched Tie2‐CYP2J2‐Tr mice (Figure 1e,f). Moreover, glucose tolerance tests indicated that aging‐related glucose intolerance was suppressed significantly in 16‐month‐old CYP2J2‐Tr mice (Figure 1g). Insulin tolerance tests also showed that 16‐month‐old CYP2J2‐Tr mice had increased insulin sensitivity when compared with 16‐month‐old WT mice (Figure 1h). No significant differences were found in these parameters between 3‐month‐old Tie2‐CYP2J2‐Tr mice (hereafter referred to as 3‐month‐old CYP2J2‐Tr mice) and their WT littermate control. Numerous studies have linked aging with deregulation of insulin signaling in multiple organs (González‐Rodríguez et al., 2012; Ropelle et al., 2013). In this study, we assessed the activation status of the insulin signaling cascade following insulin stimulation. In adipose tissue (Figure 2a,b) and muscle (Figure 2c,d), a lower level of IRβ, AKT, and GSK3β phosphorylation was observed in 16‐month‐old WT mice compared with 3‐month‐old WT mice. Endothelium‐specific CYP2J2 overexpression significantly prevented the decrease in IRβ, AKT, and GSK3β phosphorylation. These data indicated that endothelium‐specific CYP2J2 overexpression markedly attenuated aging‐related insulin resistance.

### 2.3 Aged endothelium‐specific CYP2J2 overexpression mice are protected against hepatic insulin resistance and coincide with repressed PP2A in liver

**Figure 3:**
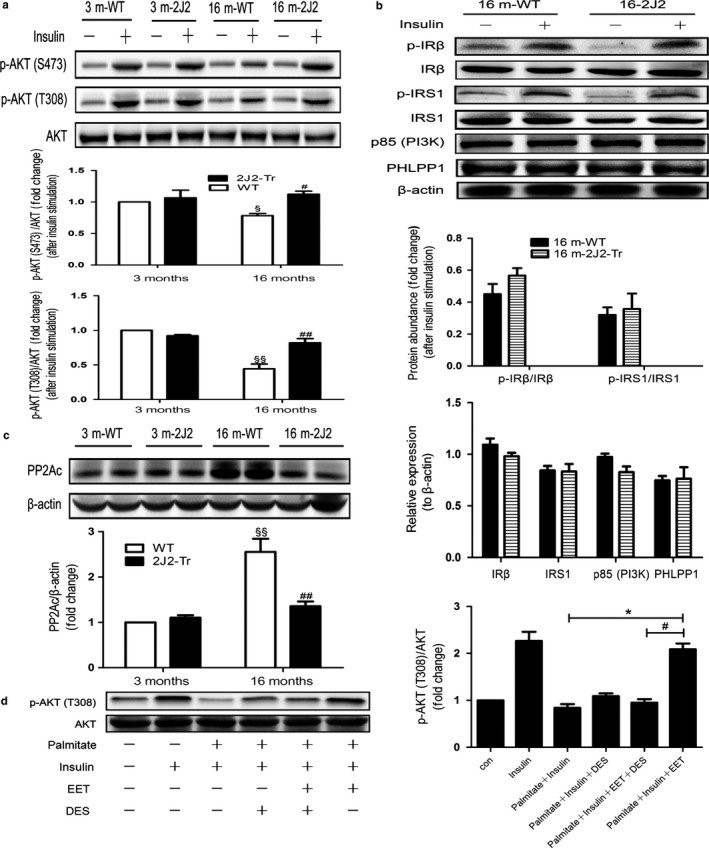
CYP2J2 overexpression‐mediated improvement in insulin sensitivity coincides with repressed PP2A in liver. Mice were anesthetized after overnight fasting, and 1 IU per kg insulin or an equal volume of vehicle was administered through the portal vein. Liver tissue was collected 120 s after the injection and analyzed by Western blot. (a) Immunoblots of p‐Akt (S473), p‐Akt (T308), and T‐Akt in liver of 3‐ or 16‐month‐old WT and 2J2‐Tr mice. (b) Liver lysates from 16‐month‐old WT and 2J2‐Tr mice were immunoblotted using the antibodies as shown. (c) Representative immunoblots and quantitation of PP2Ac and β‐actin in liver extracts. Data are shown as means ± *SE* (*n* = 6 per group). ^§^ *p *< .05, 16mWT vs. 3 mWT; ^§§^ *p *< .01, 16 mWT vs. 3 mWT; ^#^ *p *< .05, 16 m2J2‐Tr vs. 16 mWT; ^##^ *p* < .01, 16 m2J2‐Tr vs. 16 mWT. In addition, HepG2 hepatocytes (ATCC) were exposed to 0.25 mm palmitate for 24 h in the presence or absence of 10 nm D‐erythro‐sphingosine (DES) and 1 μm 14,15‐EET in serum‐free medium prior to stimulation with insulin (Sigma–Aldrich) or vehicle control (20 nm for 10 min). (d) Resulting cell lysates were immunoblotted using p‐Akt (T308) and T‐Akt antibodies. Values presented are the mean ± *SE* from three independent experiments. *p < .05, ^#^ *p *< .05

The effect of endothelium‐specific CYP2J2 overexpression on liver insulin downstream signaling pathway was assessed in this study. As expected, insulin‐stimulated AKT (S473) and AKT (T308) phosphorylations were decreased in the livers of 16‐month‐old WT mice compared with that in 3‐month‐old WT mice, and moreover, this decrease was significantly reversed in 16‐month‐old CYP2J2‐Tr mice (Figure 3a). However, as shown in Figure 3b, Tie2‐driven endothelial expression of CYP2J2 did not alter protein abundance of the p‐IRβ, p‐IRS‐1, and p85 regulatory subunit of PI3K in 16‐month‐old CYP2J2‐Tr mice compared to their WT littermates. These data demonstrated the ability of CYP2J2 overexpression to enhance insulin sensitivity in livers without altering upstream IRS‐1/PI3K signaling pathway. Therefore, we next focused on alternative pathways involving the regulatory mechanism of AKT phosphorylation. Previous study indicated that PHLPP1 and PP2A (protein phosphatase 2A) were two key negative regulators which act to dephosphorylate and inactivate AKT (Liu et al., 2012; Resjö et al., 2002). Interestingly, in this study, endothelium‐specific CYP2J2 overexpression did not alter the level of PHLPP1 protein in livers of 16‐month‐old CYP2J2‐Tr mice (Figure 3b). An aging‐related increase in liver PP2Ac protein expression was observed in 16‐month‐old WT mice, and furthermore, endothelium‐specific CYP2J2 overexpression did promote a significant reduction in the protein abundance of the PP2A catalytic subunit (PP2Ac) in the liver as shown in Figure 3c. To further explore the potential mechanism of EETs on liver phosphorylated AKT expression, HepG2 cells were used in in vitro study. Saturated fatty acids are known to induce insulin resistance in hepatic cell lines (Lee et al., 2016). Here, we made use of the palmitate‐treated HepG2 cells to investigate the effect of 14, 15‐EET on HepG2 cells phosphorylated AKT expression. As expected, palmitate treatment decreased phosphorylated AKT expression, and 14,15‐EET administration markedly prevented the decrease in phosphorylated AKT expression as shown in Figure 3d. Importantly, the regulatory effect of 14, 15‐EET on phosphorylated AKT expression was abolished by co‐treatment with DES, a potent PP2A agonist (Figure 3d). These data indicate that PP2A as a key component in age‐related insulin resistance in liver, whereby its repression in response to endothelial CYP2J2 gene targeting may serve to alleviate inactivation of AKT.

### 2.4 Endothelium‐specific CYP2J2 overexpression exerts anti‐inflammatory and anti‐adipogenic effects in adipose tissue of aged mice

**Figure 4:**
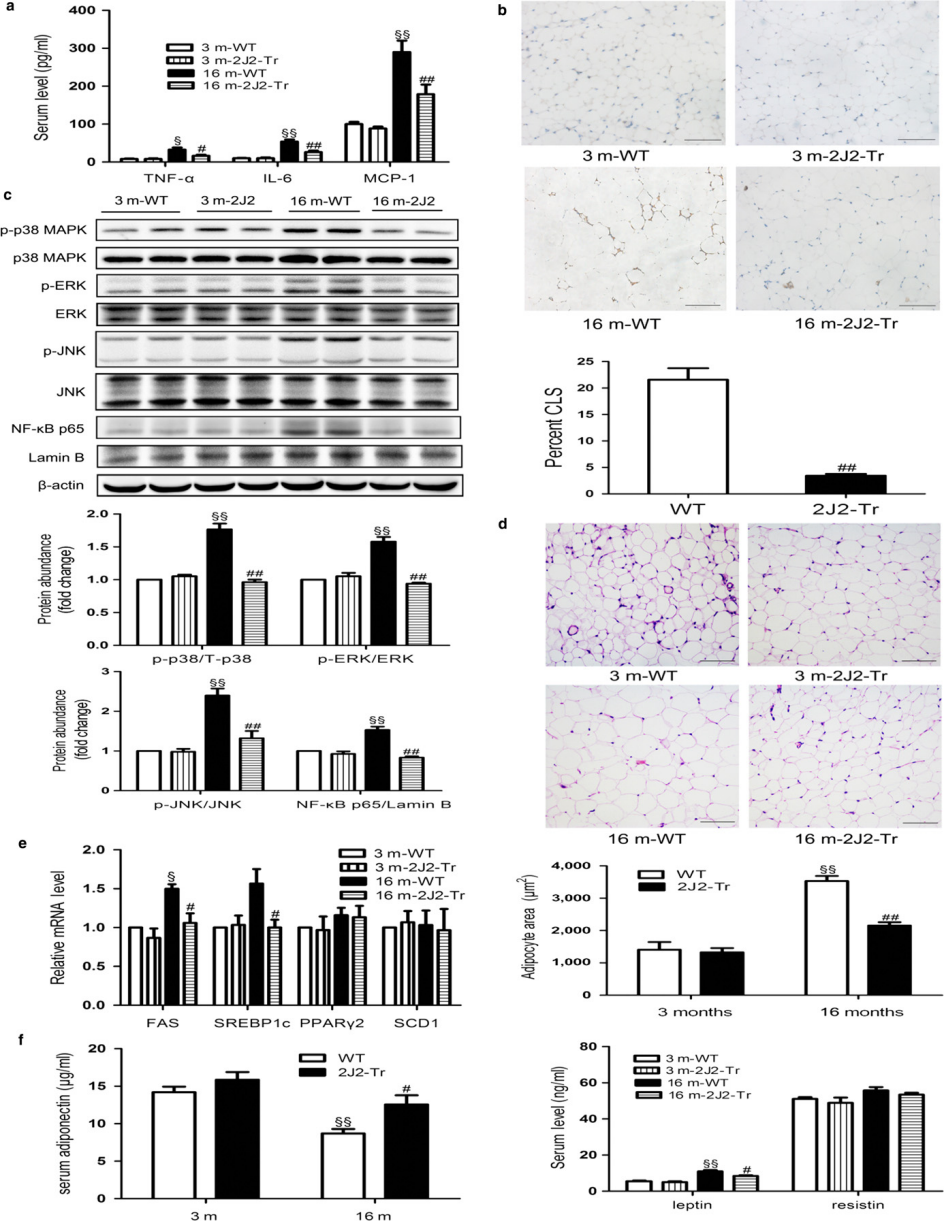
CYP2J2 overexpression conveys both anti‐inflammatory and anti‐adipogenic responses in adipose tissue of aged mice. (a) Serum levels of TNF‐α, IL‐6, and MCP‐1 were measured by ELISA. (b) Macrophage infiltration in WAT assessed by F4/80 immunostaining and the quantification of the amount of CLS (crown‐like structure) in epididymal WAT from different experimental groups of mice. Scale bars represent 100 μm. (c) Attenuation of inflammatory signaling pathways in WAT of 16 m2J2‐Tr mice. Immunoblot and quantitation of p‐p38 MAPK, p‐ERK, p‐JNK, NF‐κB p65, and their total proteins in WAT of the different experimental groups of mice. (d) Representative hematoxylin–eosin (HE) staining and quantification of adipocyte size of epididymal adipose tissue. Scale bars represent 100 μm. (e) FAS (fatty acid synthase), SREBP‐1 (sterol regulatory element binding protein‐1), PPARγ2 (peroxisome proliferator‐activated receptor γ2), and stearoyl‐CoA desaturase‐1 (SCD1) mRNA levels determined by real‐time PCR from adipose tissue. (f) Serum levels of adiponectin, leptin, and resistin as indicated from overnight‐fasted mice. Data are shown as means ± *SE* (*n* = 8 per group). ^§^ *p *< .05, 16 mWT vs. 3 mWT; ^§§^ *p *< .01, 16 mWT vs. 3 mWT; ^#^ *p *< .05, 16 m‐2J2‐Tr vs. 16 mWT; ^##^ *p *< .01, 16 m‐2J2‐Tr vs. 16 mWT

As adipose tissue accretion and inflammation may be causatively linked to insulin resistance, the potential effects of endothelium‐specific CYP2J2 overexpression on obesity‐induced inflammation associated with aging were explored in this study. The aging‐related increases in serum levels and mRNA expressions of cytokines, including TNF‐α, IL‐6, and MCP‐1, were significantly prevented in 16‐month‐old CYP2J2‐Tr mice (Figures 4a and S3). Immunohistochemical staining with antibodies against F4/80, a macrophage marker, revealed massive infiltration of macrophages into adipose tissue in 16‐month‐old WT mice, and macrophage infiltration was markedly inhibited by endothelium‐specific CYP2J2 overexpression as shown in Figure 4b. Furthermore, inflammatory signals were also attenuated in WAT of 16‐month‐old CYP2J2‐Tr mice. Phosphorylation of p38 MAPK, ERK, and JNK was decreased in aged Tie2‐CYP2J2‐Tr mice. Nuclear NF‐κB p65 level was also attenuated in 16‐month‐old CYP2J2‐Tr mice (Figure 4c). Decreased inflammatory signals resulted in improved insulin signaling as shown in Figure 2a. In addition, the increase in expression of adhesion molecules such as VCAM‐1 and E‐selectin in the aortas of aged WT mice was prevented in 16‐month‐old CYP2J2‐Tr mice. Simultaneously, eNOS expression was significantly increased in 16‐month‐old CYP2J2‐Tr mice, which may have contributed to the lower blood pressures (Figure S4).

It is now widely recognized that obesity increases the risk of developing insulin resistance (Catalano et al., 2005). Consistent with this, our body composition analysis revealed that endothelium‐specific CYP2J2 overexpression promoted a greater reduction in body fat mass in aged mice compared to aged WT littermates (Figure 1b). Histological examination of eWAT showed a direct relationship between fat accumulation and adipocyte hypertrophy. The aging‐related increases in mean adipocyte cross‐sectional area in 16‐month‐old WT mice were significantly inhibited in 16‐month‐old CYP2J2‐Tr mice (Figure 4d). We next examined the mRNA levels of the lipogenic genes in epididymal fat tissue, including FAS, SREBP‐1, PPARγ2, and SCD1. In accord with its anti‐obesity effect in older mice, an aging‐related increase in mRNA level of FAS in 16‐month‐old WT mice was observed, which was inhibited by endothelium‐specific CYP2J2 overexpression. In addition, 16‐month‐old WT mice had a tendency to have a higher SREBP1 mRNA level than younger counterparts, but this did not quite reach statistical significance. However, the mRNA level of SREBP‐1 was decreased in the 16‐month‐old CYP2J2‐Tr mice compared with that in age‐matched WT mice (Figure 4e). Furthermore, plasma levels of adiponectin were lower in 16‐month‐old WT mice vs. 3‐month‐old WT mice, but not in 16‐month‐old CYP2J2‐Tr mice (Figure 4f). The level of leptin in the 16‐month‐old WT mice was higher than that in the age‐matched Tie2‐CYP2J2‐Tr mice. However, no significant difference was seen in resistin levels between these two groups as shown in Figure 4f. Therefore, these data indicated that endothelium‐specific CYP2J2 overexpression exerted inhibitory effects on aging‐related adipose tissue inflammation and adipogenesis.

### 2.5 Effects of endothelium‐specific CYP2J2 overexpression on adipose tissue macrophage polarization

**Figure 5:**
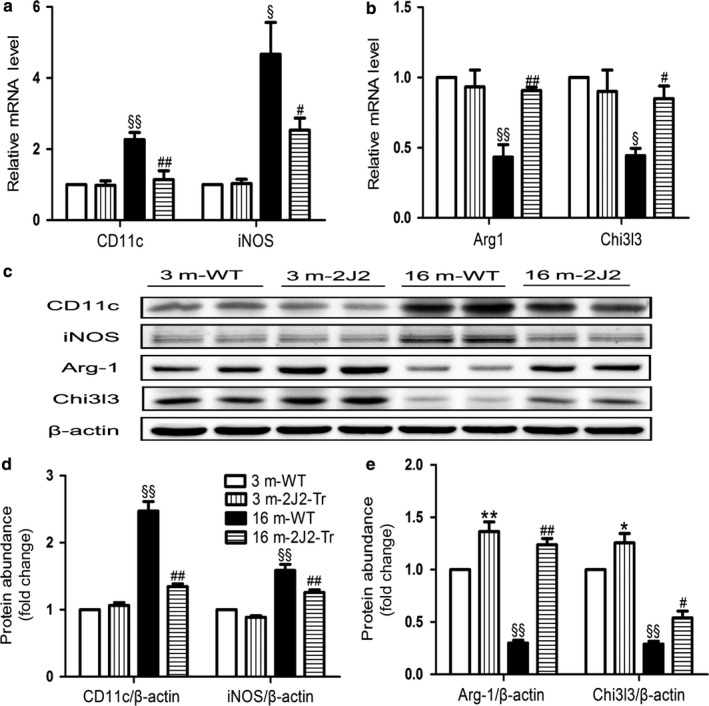
Effects of Endothelium‐specific CYP2J2 overexpression on macrophages polarization in eWAT of old mice. (a) M1 marker genes of macrophages CD11c and iNOS and (b) M2 marker genes of macrophages Arg1 and Chi3 l3 were measured by real‐time PCR. (c‐e) Representative immunoblots and quantitation of M1 (CD11c and iNOS) and M2 (Arg1 and Chi3 l3) markers. Data are shown as means ± *SE* (*n* = 8 per genotype). ^§^ *p *< .05,16 mWT vs. 3 mWT; ^§§^ *p *< .01, 16 mWT vs. 3 mWT; ^#^ *p *< .05, 16 m2J2‐Tr vs. 16 mWT; ^##^ *p *< .01, 16 m2J2‐Tr vs. 16 mWT

Consistent with the enhanced influx of macrophages into adipose tissue as determined by an immunohistochemical approach, gene expression levels of general macrophage marker F4/80 was increased in 16‐month‐old WT mice compared with that in 3‐month‐old WT mice (Figure S3). Noticeably, gene expression and serum level of the chemoattractant MCP‐1 were increased in the old WT mice, which were inhibited in age‐matched CYP2J2‐Tr mice (Figures 4a and S3). The levels of M1 markers CD11c and iNOS were lower at mRNA (Figure 5a) and protein levels (Figure 5c,d) in 16‐month‐old CYP2J2‐Tr mice compared to age‐matched WT mice. In contrast, typical markers for M2‐type macrophages, Arg1 and chi3 l3, were increased in adipose tissue of 16‐month‐old CYP2J2‐Tr mice compared with 16‐month‐old WT mice (Figure 5b,c,e). These data revealed that the immune balance within the WAT of the 16‐month‐old CYP2J2‐Tr mice was skewed to the M2 macrophages.

### 2.6 Effects of endothelium‐specific CYP2J2 overexpression on energy expenditure in 16‐month‐old mice

**Figure 6:**
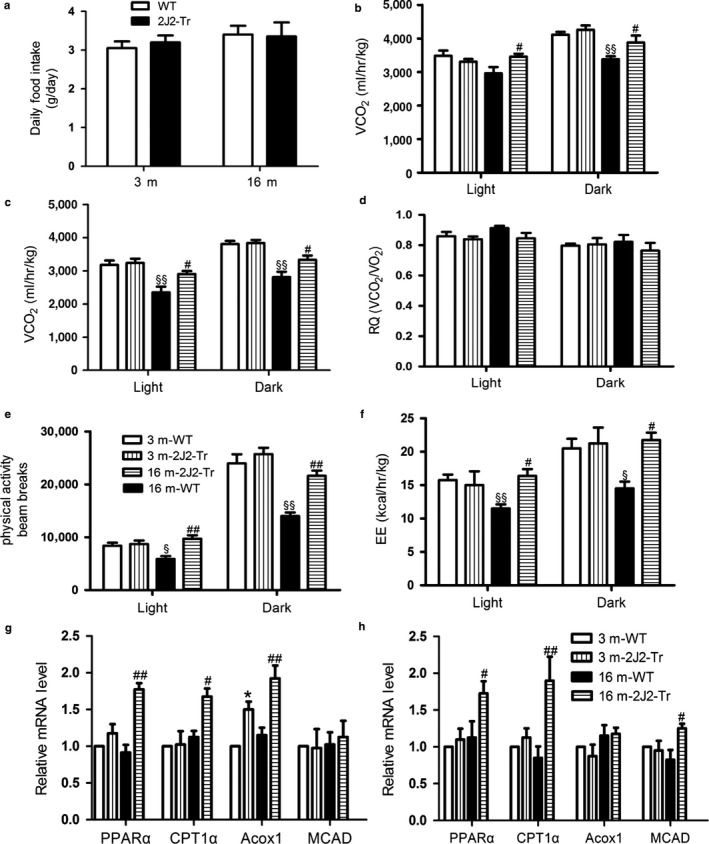
Effects of CYP2J2 overexpression on energy expenditure in 16‐month‐old mice. (a) Food intake, (b) VO2, (C) VCO_2_, (d) RQ (VCO_2_/VO_2_), (e) spontaneous activity, and (f) energy expenditure (EE) were measured by Columbus Instruments Comprehensive Lab Animal Monitoring System (CLAMS). (g) Expression of mRNA controlling fatty acid oxidation (FAO) in livers, including PPARα, carnitine palmitoyltransferase‐1α (CPT‐1α), acyl‐coenzyme A oxidase 1 (Acox1), and medium‐chain acyl‐CoA dehydrogenase (MCAD), was measured by real‐time PCR. (h) PPARα, CPT1α, Acox1, and MCAD mRNA levels determined by real‐time PCR from gastrocnemius. Data are shown as means ± *SE* (*n* = 8 per genotype). *p < .05, 3 m2J2‐Tr vs. 3 mWT; ^§^ *p *< .05, 16 mWT vs. 3 mWT; ^§§^ *p *< .01, 16 mWT vs. 3 mWT; ^#^ *p *< .05, 16 m2J2‐Tr vs. 16 mWT; ^##^ *p *< .01, 16 m2J2‐Tr vs. 16 mWT

To determine the cause of resistance to age‐related fat accumulation in 16‐month‐old CYP2J2‐Tr mice, energy expenditure parameters were examined. There were no significant differences in daily food intake between groups (Figure 6a). Furthermore, indirect calorimetry analysis showed that 16‐month‐old CYP2J2‐Tr mice consumed much greater O2 and produced more CO2 than their age‐matched control littermates during light and dark periods (Figure 6b,c). Of note, no differences between genotypes and age in respiratory quotient (RQ) values were observed, which indicated that Tie2‐CYP2J2 gene targeting does not alter fuel selection between fatty acids and carbohydrate during aging (Figure 6d). Then, we assessed whether locomotor activity was affected by endothelial CYP2J2 gene targeting. As shown in Figure 6e, 16‐month‐old WT mice exhibited a greater reduction in locomotor activity during light and dark phases compared with younger counterparts, yet in 16‐month‐old CYP2J2‐Tr mice, locomotor activity remained high compared with 16‐month‐old WT mice. Consistent with activity, energy expenditure (EE) was lower in 16‐month‐old WT mice vs. 3‐month‐old WT mice during light and dark cycles, which did not occur in 16‐month‐old CYP2J2‐Tr mice (Figure 6f). Together, these results suggest that the progressive decrease in fat mass in 16‐month‐old CYP2J2‐Tr may be due, at least in part, to increased energy expenditure.

Finally, we determined differences in expression levels of genes controlling fatty acid oxidation. In the liver (Figure 6g), there was a significant increase in PPARα, CPT1α, and Acox1 levels in 16‐month‐old CYP2J2‐Tr mice as compared with the wild‐type controls at 16 months. In skeletal muscle, PPARα, CPT1α, and MCAD were increased in 16‐month‐old CYP2J2‐Tr mice as compared with the wild‐type mice of the same age (Figure 6h). These data indicated that Tie2‐CYP2J2 promoted the process of energy expenditure at least partly via increased fatty acid oxidation.

### 2.7 Mitochondrial content and function are increased in muscles of aged endothelium‐specific CYP2J2 overexpression mice

**Figure 7:**
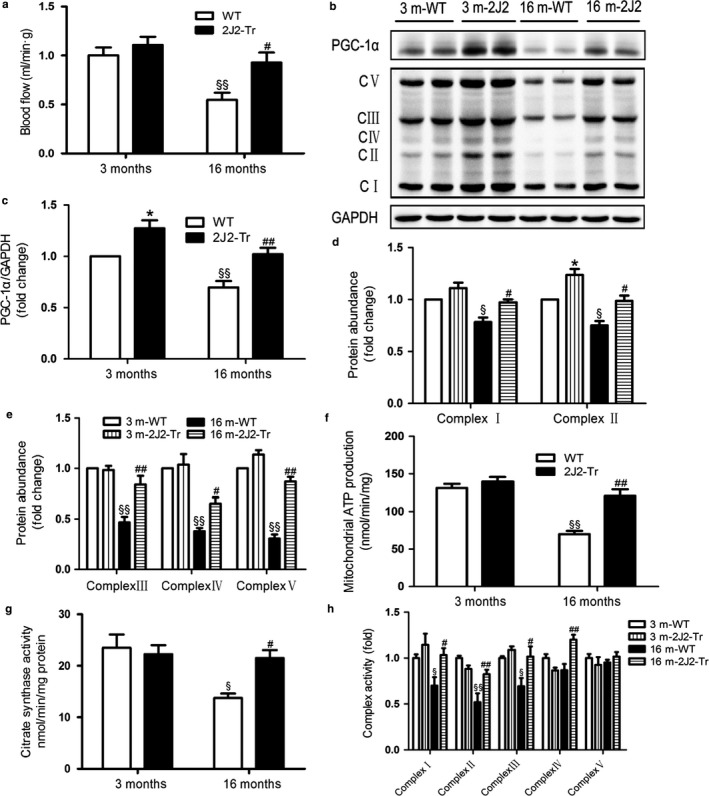
Mitochondrial content and function are increased in muscles of aged CYP2J2 overexpression mice. (a) Gastrocnemius muscle blood flows of the different experimental groups of mice were measured with the fluorescent microsphere method. (b–e) Representative immunoblots and quantitation of PGC‐1α and mitochondrial ETC. chain protein in different groups. (f) Skeletal muscle ATP levels, (g) citrate synthase (CS) activity, (h) activities of the electron transport chain (ETC.) complexes I‐V were assayed as described in MATERIALS AND METHODS. Data are shown as means ± *SE* (*n* = 8 per genotype). *p < .05, 3 m2J2‐Tr vs. 3 mWT; ^§^ *p *< .05,16 mWT vs. 3 mWT; ^§§^ *p *< .01, 16 mWT vs. 3 mWT; ^#^ *p *< .05, 16 m2J2‐Tr vs. 16 mWT; ^##^ *p *< .01, 16 m2J2‐Tr vs. 16 mWT

As Tie2‐CYP2J2 gene targeting decreased aortic vascular adhesion molecules and increased eNOS expression, respectively (Figure S4), we measured blood flow in muscle, a major insulin‐sensitive tissue, using the fluorescent microsphere method. As expected, endothelial CYP2J2 gene targeting significantly increased blood flow in the muscles of 16‐month‐old mice (Figure 7a), which suggests involvement in the prevention of age‐induced insulin resistance. Aging is also associated with reductions in skeletal muscle mitochondrial function, and mitochondrial dysfunction relates to the pathophysiology of insulin resistance in classic insulin‐responsive tissue (Kim et al., 2008; Petersen et al., 2003). Studies have shown that PGC‐1α is a master regulator of energy metabolism and mitochondrial biogenesis (Li et al., 2016; Wenz, 2013). Mitochondria are responsible for the production of cellular energy through the electron transport chain (ETC.) that consists of five protein complexes I–V. Next, we examined mitochondrial protein expression and mitochondrial function in gastrocnemius muscles of different groups. Protein expressions of PGC‐1α and complexes I–V were significantly reduced in 16‐month‐old WT mice compared with younger counterparts, suggesting aging‐associated decline in mitochondrial biogenesis. Furthermore, the increase in PGC‐1α expression in the 16‐month‐old CYP2J2‐Tr mice correlated with the increases in protein levels of all five ETC. complexes, I, II, III, IV, and V (Figure 7b–e).

We thus further focused on the effect of Tie2‐CYP2J2 gene targeting on mitochondrial function. As shown in Figure 7f, aging‐associated decreases in ATP levels in the muscle mitochondria of 16‐month‐old WT mice were inhibited in the 16‐month‐old CYP2J2‐Tr mice. We also measured citrate synthase activity (n mol/min/mg protein) in the muscle tissue. As displayed in Figure 7g, the activity of mitochondrial citrate synthase was markedly increased in 16‐month‐old CYP2J2‐Tr mice compared with their age‐matched WT group. In addition, we examined the effect of endothelium‐specific CYP2J2 overexpression on the activities of mitochondrial complexes I, II, III, IV, and V. 16‐month‐old WT mice showed reduced activities of complexes I, II, and III; however, no change in the activity of complexes IV and V was noted in comparison with 3‐month‐old WT mice. Interestingly, we observed a significant increase in activities of complexes I‐IV in muscles from 16‐month‐old CYP2J2‐Tr mice (Figure 7h). Taken together, these results suggested that the endothelium‐specific CYP2J2 overexpression led to stimulation of mitochondrial energy metabolism, which protected the mice against age‐related obesity and insulin resistance.

## 3 DISCUSSION

Insulin resistance increases with age and plays a key role in the pathogenesis of type two diabetes mellitus. Therapeutic interventions are therefore needed to improve insulin sensitivity in the elderly. In this study, the protective effects of Tie2‐CYP2J2 gene targeting in aging‐related insulin resistance were observed in 16‐month‐old mice. We demonstrated that endothelium‐specific CYP2J2 overexpression prevented the development of aging‐related insulin resistance by improving energy homeostasis, which was associated with decreased inflammatory response. Therefore, these findings revealed that CYP epoxygenase‐EETs system protected against aging‐related insulin resistance and metabolic dysfunction.

Previous studies indicated a significant reduction in EETs level in mice fed with a HF diet, which was accompanied by increased inflammatory response and oxidative stress (Sodhi et al., 2012). In the present study, we observed an aging‐related decrease in serum level of 14, 15‐EET in wild‐type mice. Consistent with previous studies, Tie2‐CYP2J2‐Tr mice displayed increased levels of plasma and tissue EETs (Abraham et al., 2014; Deng et al., 2011; Lee et al., 2010). Sterile inflammation is a common feature of aging, which is an independent risk factor for both type two diabetes and cardiovascular disease. Adipose tissue is thought to be a major contributor to the chronic, low‐grade inflammation seen in aging (Guilherme et al., 2008; Palmer & Kirkland,^2016^; Tchkonia et al., 2010). Our data showed that fat mass was increased in 16‐month‐old wild‐type mice, and moreover, endothelium‐specific CYP2J2 overexpression effectively inhibited WAT inflammation, including macrophage infiltration, adipocyte hypertrophy, and the expression of pro‐inflammatory markers in aged mice. In addition, we also examined the effect of aging on adiponectin concentrations. Adiponectin has been suggested to have both anti‐inflammatory and antidiabetic properties (Luo et al., 2010; Yamauchi et al., 2001). Our results indicated that endothelial CYP2J2 gene targeting increased the concentrations of adiponectin in 16‐month‐old CYP2J2‐Tr mice.

The aging‐related increase in adiposity has been suggested to underlie the reduced insulin sensitivity with age (Catalano et al., 2005; Karakelides et al., 2010). From middle age, the distribution of adipose tissue shifts from primarily subcutaneous depots to visceral depots, which is more closely associated with development of metabolic syndrome and insulin resistance. The location and function of adipose tissue have been suggested to be more important than the absolute amount of adipose tissue in terms of its effect on insulin sensitivity (Palmer & Kirkland, 2016; Preis et al., 2010). Inadequate angiogenic remodeling during adipose tissue expansion promotes migration of immune cells into adipose depots, resulting in adipose tissue dysfunction (Crewe, An & Scherer, 2017). In addition to the anti‐inflammatory effect, systemic administration of an EET agonist also lowers adiposity (Sodhi et al., 2009, 2012). In accord with this, our study indicated that endothelium‐specific CYP2J2 overexpression repressed aging‐associated lipogenesis, respectively, in aged epididymal fat tissue. Furthermore, the 16‐month‐old CYP2J2‐Tr mice are leaner apparently due to increased total body energy expenditure. 16‐month‐old CYP2J2‐Tr mice also showed signs of increased FAO in liver and skeletal muscle, as evidenced by higher mRNA levels of PPARα, CPT1α, Acox1, and MCAD. These may account, at least in part, for the reduction in fat depots in these mice. It is likely that the beneficial effect of EETs is primarily due to local EET production in the vasculature of the fat tissue. The mechanisms by which vascular endothelial EETs affect adipocyte function are yet to be fully explored. Hence, based on previous studies suggesting the existence of an EET receptor (Chen, Falck, Manthati, Jat & Campbell, 2011), we postulated that vascular endothelial EETs affected adipocyte function via a receptor‐mediated mechanism in an autocrine and/or paracrine fashion.

Recent study demonstrated that antimyostatin antibody increases muscle mass and strength and improves insulin sensitivity in old mice (Camporez et al., 2016). Aging‐associated sarcopenia that accompanies increased weakness and fatigability have devastating consequences for patients. Sarcopenia reduces quality of life and physical activity, and markedly increases the incidence of falls and fractures. For older people in particular, declining muscle mass causes morbidity from loss of strength, reduced energy expenditure, changes in body composition favoring increased adiposity, and increased insulin resistance (Janssen, Heymsfield, Wang & Ross, 2000; Lee et al., 2010; Piers, Soares, McCormack & O'Dea, 1998; Ryan, 2000). Our results demonstrated that endothelium‐specific CYP2J2 overexpression led to increased skeletal muscle mass and locomotor activity in aged mice. In addition, the antisarcopenic effects of CYP2J2 overexpression were associated with increased insulin‐stimulated whole‐body metabolism in the old mice. Furthermore, eNOS expression and blood flow in muscles were enhanced in 16‐month‐old CYP2J2‐Tr mice, which produced a virtuous cycle for increased microcirculation and decreased pro‐inflammatory responses (Hasegawa et al., 2012). We infer that these effects are associated with increased activity in aged mice. In contrast, the attenuation of microcirculatory blood flow observed in eNOS‐deficient mice decreases transcapillary passage of insulin to metabolically active tissues such as muscle, thereby contributing to impairment of insulin action (Kubis, Richer, Domergue, Giudicelli & Lévy, 2002). Consequently, the negative regulation of eNOS expression during aging in endothelial cells may be another important mechanism underlying insulin resistance, reduction in lean mass, and decreased locomotor activity. However, the precise mechanisms of CYP2J2 overexpression‐mediated antisarcopenic effects need to be further explored.

Aging is also associated with reductions in skeletal muscle mitochondrial function. In particular, skeletal muscle mitochondrial ATP production rates (MAPRs) are reduced in elderly people. These aging‐related reductions in MAPRs are also associated with concomitant reductions in skeletal muscle mitochondrial enzyme activities, protein synthesis and expression, and mtDNA abundance in humans and rodents (Karakelides et al., 2010; Petersen et al., 2003). Of interest, insulin resistance is closely associated with skeletal muscle mitochondrial dysfunction (Karakelides et al., 2010; Kim et al., 2008; Petersen et al., 2003). This close association between muscle mitochondrial dysfunction and insulin resistance has led to the hypothesis that mitochondrial dysfunction could be the basis of insulin resistance. Our data demonstrated that the aging‐related skeletal muscle mitochondrial dysfunction was attenuated in 16‐month‐old CYP2J2‐Tr mice. Furthermore, this attenuation occurred with increased mitochondrial biogenesis evidenced by increased mitochondrial protein expression. Others found that UA‐8, a synthetic compound that possessed both EET‐mimetic and sEH inhibitory properties, provided significant cardioprotection against ischemia–reperfusion injury via limiting mitochondrial dysfunction (Batchu et al., 2011). Some other studies showed that EETs failed to enhance mitochondrial content (Liu et al., 2011; Sarkar et al., 2014). However, the current study revealed a partial reversal of an aging‐related decline in mitochondrial biogenesis by 2J2 overexpression in the skeletal muscle of old mice. Moreover, Tie2‐CYP2J2 gene targeting had no effects on mitochondrial content and function in young mice. This apparent discrepancy between the current finding and precedent literature may be explained by the possibility that EETs may enhance mitochondrial content only under conditions where dysfunction is evident.

To summarize, endothelium‐specific EET production in male adult mice is able to counteract aging‐related metabolic disorders, particularly overweight, fat accumulation, and peripheral insulin resistance. These findings revealed the new role for EETs in conferring age‐induced insulin resistance and metabolic dysfunction, although further investigation is required to extrapolate these results into human scenarios.

## 4 EXPERIMENTAL PROCEDURES

### 4.1 Animals

All animal studies were approved by The Academy of Sciences of China and complied with standards stated in the National Institutes of Health Guidelines for the Care and Use of Laboratory Animals. Endothelium‐specific CYP2J2 overexpression mice (Tie2‐CYP2J2‐Tr) on a pure C57BL/6J genetic background were obtained from Dr. Darryl Zeldin's colony at the National Institute of Environmental Health Science (RTP, NC, USA) and were genotyped using PCR‐based methods. 3‐month‐old wild‐type (WT) littermate control mice (3 mWT), 3‐month‐old Tie2‐CYP2J2‐Tr (3m2J2‐Tr), 16‐month‐old WT littermate control mice (16 mWT), and 16‐month‐old Tie2‐CYP2J2‐Tr mice (16 m2J2‐Tr) were used in this study. Mice were fed with a standard chow diet ad libitum and had free access to drinking water.

### 4.2 Evaluation of in vivo insulin signaling

After overnight fasting, mice were anesthetized and 1 IU per kg human insulin (Novo Nordisk, Novo Alle′, Denmark) or an equal volume of vehicle was administered through the portal vein. Adipose tissue (epididymal fat pads), liver, and gastrocnemius muscle were collected 120 s after the injection and immediately stored in liquid nitrogen for subsequent Western blotting analysis (Law et al., 2010).

### 4.3 Quantitative real‐time PCR

Total RNA extracts were isolated from tissue lysate using TRIzol reagent (Invitrogen, USA) following the manufacturer's instructions. Total RNA was reversely transcribed using the TransScript First‐Strand cDNA Synthesis kit, and quantitative real‐time PCR was performed on an ABI7900 PCR system (Applied Biosystems, Darmstadt, Germany) using the TransStart^TM^ Eco Green qPCR Kit (Qiagen, Valencia, CA, USA). Primers used for real‐time PCR are shown in Table 1.

**Table 1:** Primer sequences for quantitative real‐time PCR

Gene	Forward primer	Reverse primer
F4/80	CTTTGGCTATGGGCTTCCAGTC	GCAAGGAGGACAGAGTTTATCGTG
TNF‐α	CATCTTCTCAAAATTCGAGTGACAA	TGGGAGTAGACAAGGTACAACCC
IL‐6	GGACCAAGACCATCCAATTCATCTTGAAA	GACCACAGTGAGGAATGTCCACAAA
MCP‐1	TTAAAAACCTGGATCGGAACCAA	GCATTAGCTTCAGATTTACGGGT
FAS	AGAGACGTGTCACTCCTGGACTT	GCTGCGGAAACTTCAGAAAAT
SREBP1c	GGAGCCATGGATTGCACATT	GGCCCGGGAAGTCACTGT
PPARγ2	GCTGCAGCGCTAAATTCATCT	GGGAGTGGTCATCCATCACAG
SCD1	CCGGAGACCCCTTAGATCGA	TAGCCTGTAAAGATTTCTGCAAACC
Arg‐1	CTCCAAGCCAAAGTCCTTAGAG	AGGAGCTGTCATTAGGGACATC
Chi3 l3	AGAAGGGAGTTTCAAACCTGGT	GTCTTGCTCATGTGTGTAAGT GA
CD11c	GCTGTCTCCAAGTTGCTCAGA	GAGCACACTGTGTCCGAACT
iNOS	CCAAGCCCTCACCTACTTCC	CTCTGAGGGCTGACACAAGG
PPARα	GAGGGTTGAGCTCAGTCAGG	GGTCACCTACGAGTGGCATT
CPT1α	ACTCCTGGAAGAAGAAGTTCAT	AGTATCTTTGACAGCTGGGAC
Acox1	GGGAGTGCTACGGGTTACATG	CCGATATCCCCAACAGTGATG
MCAD	TGGCATATGGGTGTACAGGG	CCAAATACTTCTTCTTCTGTTGATCA
β‐actin	AGGCCCAGAGCAAGAGAGGTA	GGGGTGTTGAAGGTCTCAAACA

### 4.4 Insulin and Glucose Tolerance Tests

For glucose tolerance tests (GTTs), mice were injected intraperitoneally with 2 g/kg glucose after a 16‐hr fasting. Blood glucose levels were measured immediately before and 30, 60, 90, and 120 min after glucose injection. Insulin tolerance tests (ITTs) were performed on 5‐hr‐fasted mice injected intraperitoneally with 0.75 U/kg human insulin (Novo Nordisk, Novo Alle′, Denmark). Blood glucose levels were determined as described above. Area under the curve (AUC) values were calculated with the GraphPad Prism software (GraphPad Software Inc.).

### 4.5 Indirect calorimetry analysis

In vivo metabolic assessment via indirect calorimetry was performed using the Oxymax CLAMS (Columbus Instruments, Columbus, OH, USA) with an airflow of 0.6 L/min and sample flow of 0.5 L/min. Mice were individually housed in metabolic chambers maintained at 20–22°C in a 12‐hr light/12‐hr dark cycle. After the mice had adapted to the environment of the metabolic chamber for 48 hr, the subsequent 24 hr period was utilized for data collection.

### 4.6 Statistical analysis

All values are presented as mean ± *SEM*. The in vivo data were analyzed using Student's unpaired *t* test or two‐way ANOVA with Bonferroni's multiple comparison post‐test. The in vitro data were analyzed by one‐way ANOVA followed by the Tukey's multiple comparison post hoc test. Statistical calculations were performed using the GraphPad Prism software (GraphPad, San Diego, CA). *p* values of <.05 were considered significant.

All other methods are described in the Appendix S1.

## ACKNOWLEDGMENTS

We are deeply grateful to Dr. Darryl Zeldin for providing the WT and endothelium‐specific CYP2J2 overexpression (Tie2‐CYP2J2‐Tr) mice.

## CONFLICT OF INTEREST

The authors declare that they have no conflict of interest.

## AUTHOR CONTRIBUTIONS

Y.Y., D.W., X.X. and L.T. designed research; Y.Y., Z.C., D.H., M.F. and Y.T. performed research; Y.Y., R.D. and L.T. analyzed data; and Y.Y. and X.X. wrote the manuscript.

## References

[acel12718-bib-0001] Abraham, N. G. , Sodhi, K. , Silvis, A. M. , Vanella, L. , Favero, G. , Rezzani, R. , … Schwartzman, M. L. (2014). CYP2J2 targeting to endothelial cells attenuates adiposity and vascular dysfunction in mice fed a high‐fat diet by reprogramming adipocyte phenotype. Hypertension, 64, 1352–1361. https://doi.org/10.1161/HYPERTENSIONAHA.114.03884 25245389 10.1161/HYPERTENSIONAHA.114.03884 PMC4230994

[acel12718-bib-0002] Akasaki, Y. , Ouchi, N. , Izumiya, Y. , Bernardo, B. L. , Lebrasseur, N. K. , & Walsh, K. (2014). Glycolytic fast‐twitch muscle fiber restoration counters adverse age‐related changes in body composition and metabolism. Aging Cell, 13, 80–91. https://doi.org/10.1111/acel.12153 24033924 10.1111/acel.12153 PMC3947044

[acel12718-bib-0003] Barbieri, M. , Rizzo, M. R. , Manzella, D. , & Paolisso, G. (2001). Age‐related insulin resistance: Is it an obligatory finding? The lesson from healthy centenarians. Diabetes/Metabolism Research and Reviews, 17, 19–26. https://doi.org/10.1002/(ISSN)1520-7560 11241888 10.1002/dmrr.178

[acel12718-bib-0004] Batchu, S. N. , Lee, S. B. , Qadhi, R. S. , Chaudhary, K. R. , El‐Sikhry, H. , Kodela, R. , … Seubert, J. M. (2011). Cardioprotective effect of a dual acting epoxyeicosatrienoic acid analogue towards ischaemia reperfusion injury. British Journal of Pharmacology, 162, 897–907. https://doi.org/10.1111/j.1476-5381.2010.01093.x 21039415 10.1111/j.1476-5381.2010.01093.x PMC3042200

[acel12718-bib-0005] Burgess, A. , Vanella, L. , Bellner, L. , Schwartzman, M. L. , & Abraham, N. G. (2012). Epoxyeicosatrienoic acids and heme oxygenase‐1 interaction attenuates diabetes and metabolic syndrome complications. Prostaglandins & Other Lipid Mediators, 97, 1–16. https://doi.org/10.1016/j.prostaglandins.2011.10.002 22100745 10.1016/j.prostaglandins.2011.10.002 PMC3261364

[acel12718-bib-0006] Camporez, J. P. , Petersen, M. C. , Abudukadier, A. , Moreira, G. V. , Jurczak, M. J. , Friedman, G. , … Shulman, G. I. (2016). Anti‐myostatin antibody increases muscle mass and strength and improves insulin sensitivity in old mice. Proceedings of the National Academy of Sciences of the United States of America, 113, 2212–2217. https://doi.org/10.1073/pnas.1525795113 26858428 10.1073/pnas.1525795113 PMC4776508

[acel12718-bib-0007] Catalano, K. J. , Bergman, R. N. , & Ader, M. (2005). Increased susceptibility to insulin resistance associated with abdominal obesity in aging rats. Obesity Research, 13, 11–20. https://doi.org/10.1038/oby.2005.4 15761159 10.1038/oby.2005.4

[acel12718-bib-0008] Chen, Y. , Falck, J. R. , Manthati, V. L. , Jat, J. L. , & Campbell, W. B. (2011). 20‐Iodo‐14,15‐epoxyeicosa‐8(Z)‐enoyl‐3‐azidophenylsulfonamide: Photoaffinity labeling of a 14,15‐epoxyeicosatrienoic acid receptor. Biochemistry, 50, 3840–3848. https://doi.org/10.1021/bi102070w 21469660 10.1021/bi102070w PMC3100183

[acel12718-bib-0009] Crewe, C. , An, Y. A. , & Scherer, P. E. (2017). The ominous triad of adipose tissue dysfunction: Inflammation, fibrosis, and impaired angiogenesis. The Journal of Clinical Investigation, 127, 74–82. https://doi.org/10.1172/JCI88883 28045400 10.1172/JCI88883 PMC5199684

[acel12718-bib-0010] Deng, Y. , Edin, M. L. , Theken, K. N. , Schuck, R. N. , Flake, G. P. , Kannon, M. A. , … Lee, C. R. (2011). Endothelial CYP epoxygenase overexpression and soluble epoxide hydrolase disruption attenuate acute vascular inflammatory responses in mice. FASEB Journal, 25, 703–713. https://doi.org/10.1096/fj.10-171488 21059750 10.1096/fj.10-171488 PMC3023387

[acel12718-bib-0011] Ford, E. S. , Giles, W. H. , & Dietz, W. H. (2002). Prevalence of the metabolic syndrome among US adults: Findings from the third National Health and Nutrition Examination Survey. JAMA, 287, 356–359. https://doi.org/10.1001/jama.287.3.356 11790215 10.1001/jama.287.3.356

[acel12718-bib-0012] González‐Rodríguez, A. , Más‐Gutierrez, J. A. , Mirasierra, M. , Fernandez‐Pérez, A. , Lee, Y. J. , Ko, H. J. , … Valverde, A. M. (2012). Essential role of protein tyrosine phosphatase 1B in obesity‐induced inflammation and peripheral insulin resistance during aging. Aging Cell, 11, 284–296. https://doi.org/10.1111/j.1474-9726.2011.00786.x 22221695 10.1111/j.1474-9726.2011.00786.x PMC3306541

[acel12718-bib-0013] Guilherme, A. , Virbasius, J. V. , Puri, V. , & Czech, M. P. (2008). Adipocyte dysfunctions linking obesity to insulin resistance and type 2 diabetes. Nature Reviews Molecular Cell Biology, 9, 367–377. https://doi.org/10.1038/nrm2391 18401346 10.1038/nrm2391 PMC2886982

[acel12718-bib-0014] Hasegawa, Y. , Saito, T. , Ogihara, T. , Ishigaki, Y. , Yamada, T. , Imai, J. , … Katagiri, H. (2012). Blockade of the nuclear factor‐κB pathway in the endothelium prevents insulin resistance and prolongs life spans. Circulation, 125, 1122–1133. https://doi.org/10.1161/CIRCULATIONAHA.111.054346 22302838 10.1161/CIRCULATIONAHA.111.054346

[acel12718-bib-0016] Janssen, I. , Heymsfield, S. B. , Wang, Z. M. , & Ross, R. (2000). Skeletal muscle mass and distribution in 468 men and women aged 18‐88 yr. Journal of Applied Physiology, 89, 81–88. 10904038 10.1152/jappl.2000.89.1.81

[acel12718-bib-0017] Karakelides, H. , Irving, B. A. , Short, K. R. , O'Brien, P. , & Nair, K. S. (2010). Age, obesity, and sex effects on insulin sensitivity and skeletal muscle mitochondrial function. Diabetes, 59, 89–97. https://doi.org/10.2337/db09-0591 19833885 10.2337/db09-0591 PMC2797949

[acel12718-bib-0018] Kim, J. A. , Wei, Y. , & Sowers, J. R. (2008). Role of mitochondrial dysfunction in insulin resistance. Circulation Research, 102, 401–414. https://doi.org/10.1161/CIRCRESAHA.107.165472 18309108 10.1161/CIRCRESAHA.107.165472 PMC2963150

[acel12718-bib-0019] Kubis, N. , Richer, C. , Domergue, V. , Giudicelli, J. F. , & Lévy, B. I. (2002). Role of microvascular rarefaction in the increased arterial pressure in mice lacking for the endothelial nitric oxide synthase gene (eNOS3pt‐/‐). Journal of Hypertension, 20, 1581–1587. https://doi.org/10.1097/00004872-200208000-00021 12172320 10.1097/00004872-200208000-00021

[acel12718-bib-0021] Law, I. K. , Xu, A. , Lam, K. S. , Berger, T. , Mak, T. W. , Vanhoutte, P. M. , … Wang, Y. (2010). Lipocalin‐2 deficiency attenuates insulin resistance associated with aging and obesity. Diabetes, 59, 872–882. https://doi.org/10.2337/db09-1541 20068130 10.2337/db09-1541 PMC2844835

[acel12718-bib-0022] Lee, C. R. , Imig, J. D. , Edin, M. L. , Foley, J. , DeGraff, L. M. , Bradbury, J. A. , … Zeldin, D. C. (2010). Endothelial expression of human cytochrome P450 epoxygenases lowers blood pressure and attenuates hypertension‐induced renal injury in mice. FASEB Journal, 24, 3770–3781. https://doi.org/10.1096/fj.10-160119 20495177 10.1096/fj.10-160119 PMC2996903

[acel12718-bib-0023] Lee, G. H. , Oh, K. J. , Kim, H. R. , Han, H. S. , Lee, H. Y. , Park, K. G. , … Chae, H. J. (2016). Effect of BI‐1 on insulin resistance through regulation of CYP2E1. Scientific Reports, 6, 32229 https://doi.org/10.1038/srep32229 27576594 10.1038/srep32229 PMC5006057

[acel12718-bib-0024] Li, R. , Xu, X. , Chen, C. , Wang, Y. , Gruzdev, A. , Zeldin, D. C. , & Wang, D. W. (2015). CYP2J2 attenuates metabolic dysfunction in diabetic mice by reducing hepatic inflammation via the PPARγ. American Journal of Physiology. Endocrinology and Metabolism, 308, E270–E282. https://doi.org/10.1152/ajpendo.00118.2014 25389363 10.1152/ajpendo.00118.2014 PMC4329496

[acel12718-bib-0025] Li, Y. , Xu, S. , Li, J. , Zheng, L. , Feng, M. , Wang, X. , … Liang, P. (2016). SIRT1 facilitates hepatocellular carcinoma metastasis by promoting PGC‐1α‐mediated mitochondrial biogenesis. Oncotarget, 7, 29255–29274. https://doi.org/10.18632/oncotarget.8711 27081083 10.18632/oncotarget.8711 PMC5045394

[acel12718-bib-0026] Liu, L. , Chen, C. , Gong, W. , Li, Y. , Edin, M. L. , Zeldin, D. C. , & Wang, D. W. (2011). Epoxyeicosatrienoic acids attenuate reactive oxygen species level, mitochondrial dysfunction, caspase activation, and apoptosis in carcinoma cells treated with arsenic trioxide. Journal of Pharmacology and Experimental Therapeutics, 339, 451–463. https://doi.org/10.1124/jpet.111.180505 21846841 10.1124/jpet.111.180505 PMC3199997

[acel12718-bib-0027] Liu, J. , Zhou, L. , Xiong, K. , Godlewski, G. , Mukhopadhyay, B. , Tam, J. , … Kunos, G. (2012). Hepatic cannabinoid receptor‐1 mediates diet‐induced insulin resistance via inhibition of insulin signaling and clearance in mice. Gastroenterology, 142(1218–1228), e1. 10.1053/j.gastro.2012.01.032 PMC3482511 22307032

[acel12718-bib-0028] Luo, N. , Liu, J. , Chung, B. H. , Yang, Q. , Klein, R. L. , Garvey, W. T. , & Fu, Y. (2010). Macrophage adiponectin expression improves insulin sensitivity and protects against inflammation and atherosclerosis. Diabetes, 59, 791–799. https://doi.org/10.2337/db09-1338 20350970 10.2337/db09-1338 PMC2844826

[acel12718-bib-0029] Palmer, A. K. , & Kirkland, J. L. (2016). Aging and adipose tissue: Potential interventions for diabetes and regenerative medicine. Experimental Gerontology, 86, 97–105. https://doi.org/10.1016/j.exger.2016.02.013 26924669 10.1016/j.exger.2016.02.013 PMC5001933

[acel12718-bib-0030] Petersen, K. F. , Befroy, D. , Dufour, S. , Dziura, J. , Ariyan, C. , Rothman, D. L. , … Shulman, G. I. (2003). Mitochondrial dysfunction in the elderly: Possible role in insulin resistance. Science, 300, 1140–1142. https://doi.org/10.1126/science.1082889 12750520 10.1126/science.1082889 PMC3004429

[acel12718-bib-0031] Piers, L. S. , Soares, M. J. , McCormack, L. M. , & O'Dea, K. (1998). Is there evidence for an age‐related reduction in metabolic rate? Journal of Applied Physiology, 85, 2196–2204. 9843543 10.1152/jappl.1998.85.6.2196

[acel12718-bib-0032] Preis, S. R. , Massaro, J. M. , Robins, S. J. , Hoffmann, U. , Vasan, R. S. , Irlbeck, T. , … Fox, C. S. (2010). Abdominal subcutaneous and visceral adipose tissue and insulin resistance in the Framingham heart study. Obesity (Silver Spring), 18, 2191–2198. https://doi.org/10.1038/oby.2010.59 20339361 10.1038/oby.2010.59 PMC3033570

[acel12718-bib-0033] Resjö, S. , Göransson, O. , Härndahl, L. , Zolnierowicz, S. , Manganiello, V. , & Degerman, E. (2002). Protein phosphatase 2A is the main phosphatase involved in the regulation of protein kinase B in rat adipocytes. Cellular Signalling, 14, 231–238. https://doi.org/10.1016/S0898-6568(01)00238-8 11812651 10.1016/s0898-6568(01)00238-8

[acel12718-bib-0034] Ropelle, E. R. , Pauli, J. R. , Cintra, D. E. , da Silva, A. S. , De Souza, C. T. , Guadagnini, D. , … Carvalheira, J. B. (2013). Targeted disruption of inducible nitric oxide synthase protects against aging, S‐nitrosation, and insulin resistance in muscle of male mice. Diabetes, 62, 466–470. https://doi.org/10.2337/db12-0339 22991447 10.2337/db12-0339 PMC3554348

[acel12718-bib-0035] Ryan, A. S. (2000). Insulin resistance with aging: Effects of diet and exercise. Sports Medicine (Auckland, N. Z.), 30, 327–346. https://doi.org/10.2165/00007256-200030050-00002 10.2165/00007256-200030050-00002 11103847

[acel12718-bib-0036] Sarkar, P. , Zaja, I. , Bienengraeber, M. , Rarick, K. R. , Terashvili, M. , Canfield, S. , … Harder, D. R. (2014). Epoxyeicosatrienoic acids pretreatment improves amyloid β‐induced mitochondrial dysfunction in cultured rat hippocampal astrocytes. American Journal of Physiology. Heart and Circulatory Physiology, 306, H475–H484. https://doi.org/10.1152/ajpheart.00001.2013 24285116 10.1152/ajpheart.00001.2013 PMC3920242

[acel12718-bib-0037] Singh, S. P. , Bellner, L. , Vanella, L. , Cao, J. , Falck, J. R. , Kappas, A. , & Abraham, N. G. (2016). Downregulation of PGC‐1α prevents the beneficial effect of EET‐Heme Oxygenase‐1 on mitochondrial integrity and associated metabolic function in obese mice. Journal of Nutrition and Metabolism, 2016, 9039754. 28097021 10.1155/2016/9039754 PMC5206458

[acel12718-bib-0038] Singh, S. P. , Schragenheim, J. , Cao, J. , Falck, J. R. , Abraham, N. G. , & Bellner, L. (2016). PGC‐1 alpha regulates HO‐1 expression, mitochondrial dynamics and biogenesis: Role of epoxyeicosatrienoic acid. Prostaglandins & Other Lipid Mediators, 125, 8–18. https://doi.org/10.1016/j.prostaglandins.2016.07.004 27418542 10.1016/j.prostaglandins.2016.07.004 PMC5536246

[acel12718-bib-0039] Sodhi, K. , Inoue, K. , Gotlinger, K. H. , Canestraro, M. , Vanella, L. , Kim, D. H. , … Abraham, N. G. (2009). Epoxyeicosatrienoic acid agonist rescues the metabolic syndrome phenotype of HO‐2‐null mice. Journal of Pharmacology and Experimental Therapeutics, 331, 906–916. https://doi.org/10.1124/jpet.109.157545 19717790 10.1124/jpet.109.157545 PMC2784709

[acel12718-bib-0040] Sodhi, K. , Puri, N. , Inoue, K. , Falck, J. R. , Schwartzman, M. L. , & Abraham, N. G. (2012). EET agonist prevents adiposity and vascular dysfunction in rats fed a high fat diet via a decrease in Bach 1 and an increase in HO‐1 levels. Prostaglandins & Other Lipid Mediators, 98, 133–142. https://doi.org/10.1016/j.prostaglandins.2011.12.004 22209722 10.1016/j.prostaglandins.2011.12.004 PMC3449325

[acel12718-bib-0042] Tchkonia, T. , Morbeck, D. E. , Von Zglinicki, T. , Van Deursen, J. , Lustgarten, J. , Scrable, H. , … Kirkland, J. L. (2010). Fat tissue, aging, and cellular senescence. Aging Cell, 9, 667–684. https://doi.org/10.1111/j.1474-9726.2010.00608.x 20701600 10.1111/j.1474-9726.2010.00608.x PMC2941545

[acel12718-bib-0043] Theken, K. N. , Deng, Y. , Schuck, R. N. , Oni‐Orisan, A. , Miller, T. M. , Kannon, M. A. , … Lee, C. R. (2012). Enalapril reverses high‐fat diet‐induced alterations in cytochrome P450‐mediated eicosanoid metabolism. American Journal of Physiology. Endocrinology and Metabolism, 302, E500–E509. https://doi.org/10.1152/ajpendo.00370.2011 22185841 10.1152/ajpendo.00370.2011 PMC3311291

[acel12718-bib-0044] Wenz, T. (2013). Regulation of mitochondrial biogenesis and PGC‐1α under cellular stress. Mitochondrion, 13, 134–142. https://doi.org/10.1016/j.mito.2013.01.006 23347985 10.1016/j.mito.2013.01.006

[acel12718-bib-0045] Xu, X. , Zhang, X. A. , & Wang, D. W. (2011). The roles of CYP450 epoxygenases and metabolites, epoxyeicosatrienoic acids, in cardiovascular and malignant diseases. Advanced Drug Delivery Reviews, 63, 597–609. https://doi.org/10.1016/j.addr.2011.03.006 21477627 10.1016/j.addr.2011.03.006

[acel12718-bib-0046] Xu, X. , Zhao, C. X. , Wang, L. , Tu, L. , Fang, X. , Zheng, C. , … Wang, D. W. (2010). Increased CYP2J3 expression reduces insulin resistance in fructose‐treated rats and db/db mice. Diabetes, 59, 997–1005. https://doi.org/10.2337/db09-1241 20068141 10.2337/db09-1241 PMC2844847

[acel12718-bib-0047] Yamauchi, T. , Kamon, J. , Waki, H. , Terauchi, Y. , Kubota, N. , Hara, K. , … Kadowaki, T. (2001). The fat‐derived hormone adiponectin reverses insulin resistance associated with both lipoatrophy and obesity. Nature Medicine, 7, 941–946. https://doi.org/10.1038/90984 10.1038/90984 11479627

[acel12718-bib-0048] Zhang, S. , Chen, G. , Li, N. , Dai, M. , Chen, C. , Wang, P. , … Xu, X. (2015). CYP2J2 overexpression ameliorates hyperlipidemia via increased fatty acid oxidation mediated by the AMPK pathway. Obesity (Silver Spring), 23, 1401–1413. https://doi.org/10.1002/oby.21115 26053032 10.1002/oby.21115 PMC4565055

