# Supplementary figures and images for: Endothelium‐specific CYP2J2 overexpression attenuates age‐related insulin resistance
Source: Aging Cell. 2018 Jan 10;17(2):e12718. doi: 10.1111/acel.12718 (PMC5847864; doi:10.1111/acel.12718)

supplement figure 1


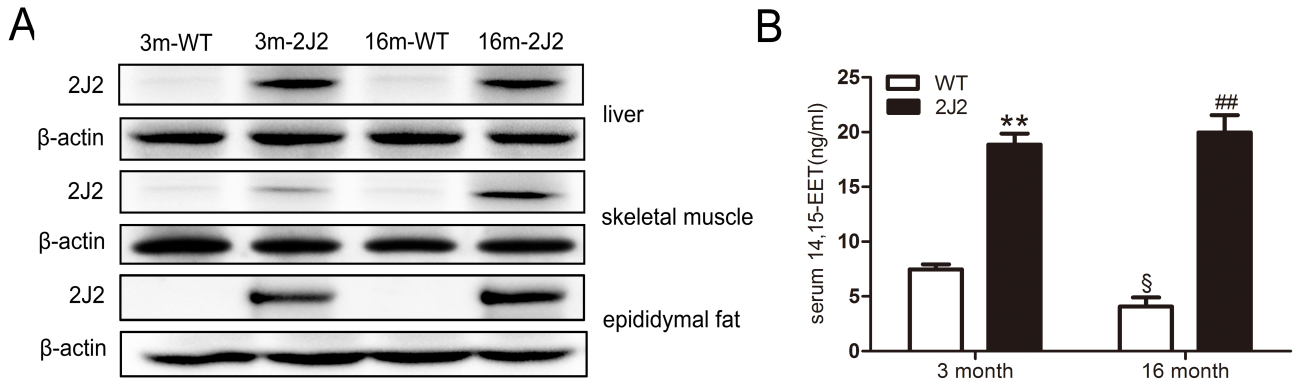


supplement figure 2


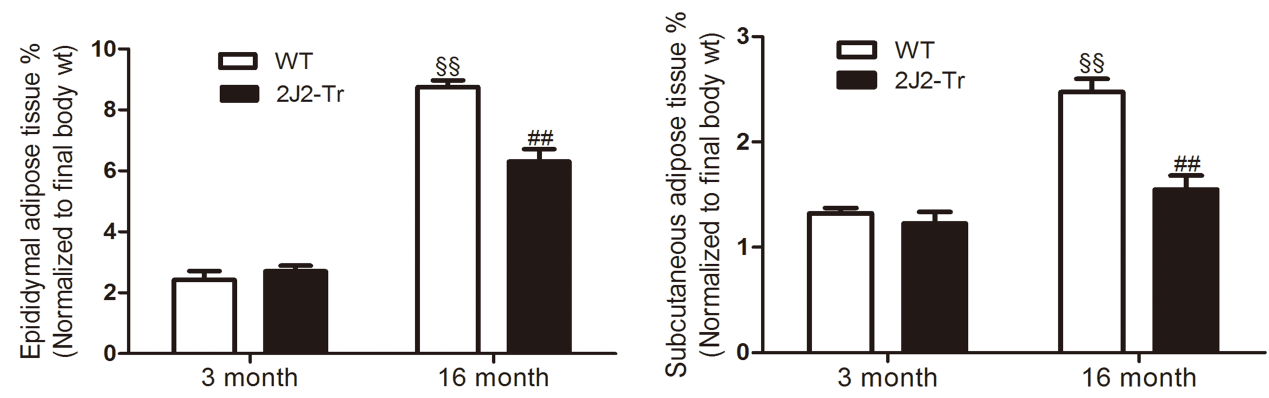


supplement figure 3


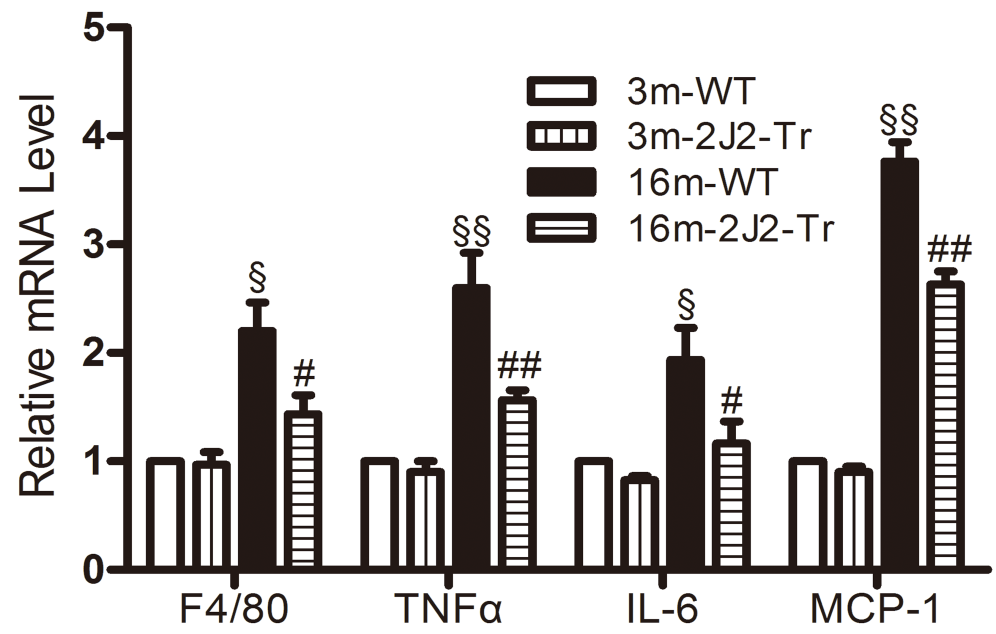


supplement figure 4


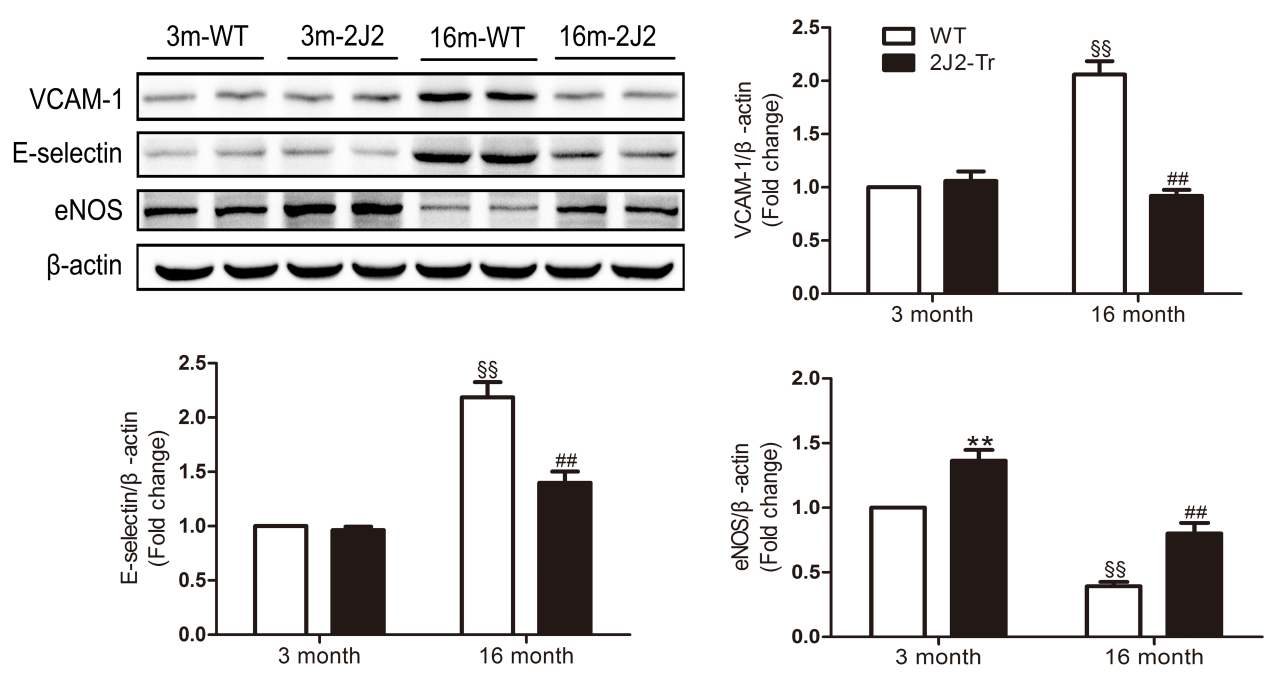

Supplement: Supplementary file 1 [file ACEL-17-e12718-s001.doc]
