# Supplementary material for: Endothelium‐specific CYP2J2 overexpression attenuates age‐related insulin resistance
Source: Aging Cell. 2018 Jan 10;17(2):e12718. doi: 10.1111/acel.12718 (PMC5847864; doi:10.1111/acel.12718)
Supplement: Supplementary file 2 [file ACEL-17-e12718-s002.doc]

**Supplementary Figure 1. Expression of CYP2J2 in liver, skeletal muscle and WAT and detection of 14,15-EET (14,15-epoxyeicosatrienoic acid) in serum.**

(A) Representative CYP2J2 protein expression levels in tissues from the 4 experimental groups. (B) 14,15-EET levels in serum. Data are shown as means ± SE (n=6 per group). ^**^*P*＜0.01, 3m-2J2 vs. 3m-WT; ^§^*P*＜0.05，16m-WT vs. 3m-WT; ^##^*P*＜0.01, 16m-2J2 vs. 16m-WT.

**Supplementary Figure 2. Effect of CYP2J2 overexpression on body compositions in young and aged mice.**

Epididymal and subcutaneous fat was measured. Data are shown as means ± SE (n=9 per group). ^§§^*P*＜0.01, 16m-WT vs. 3m-WT; ^##^*P*＜0.01, 16m-2J2 vs. 16m-WT.

**Supplementary Figure 3. CYP2J2 overexpression decreases inflammatory cytokine mRNA expression in adipose tissue during aging.**

RT-PCR analysis of the transcript levels of F4/80, TNF-α, IL-6 and MCP-1 in epididymal WAT. Data are shown as means ± SE (n=5 per group). ^§^*P*＜0.05，16m-WT vs. 3m-WT; ^§§^*P*＜0.01, 16m-WT vs. 3m-WT; ^#^*P*＜0.05, 16m-2J2 vs. 16m-WT; ^##^*P*＜0.01, 16m-2J2 vs. 16m-WT.

**Supplementary Figure 4. CYP2J2 overexpression regulates protein expression of vascular cell adhesion molecule-1 (VCAM-1), E-selectin, and endothelial nitric oxide synthase (eNOS) in aorta during aging.**

Representative immunoblot and quantitation of VCAM-1, E-selectin, eNOS and β-actin in aorta from the 4 experimental groups. Data are shown as means ± SE (n=5 per group). ^**^*P*＜0.01, 3m-2J2 vs. 3m-WT; ^§§^*P*＜0.01, 16m-WT vs. 3m-WT; ^##^*P*＜0.01, 16m-2J2 vs. 16m-WT.

**Appendix S1 Methods.**

**Reagents**

D-erythro-sphingosine (DES) (10 nM for cell culture) was purchased from Calbiochem (San Diego, CA, USA). Palmitate was purchased from Sigma-Aldrich (St Louis, MO, USA) and all other chemicals were obtained from Sigma–Aldrich unless otherwise indicated. Total OXPHOS Rodent WB Antibody Cocktail, anti-E-selectin, eNOS, PGC-1α and iNOS antibodies were purchased from Abcam (Cambridge, UK). Anti-VCAM-1, F4/80, PP2Ac, Arginase-1, β-actin, Lamin B1 and p-Irβ (Tyr1162/1163) antibodies were purchased from Santa Cruz Biotechnology (Santa Cruz, CA). Anti-p-GSK3β (ser9), GSK3β, p85 (PI3K), IRS1, p-IRS1 (Ser307), T-Akt, p-Akt (Ser473), p-Akt (Thr308), IRβ, p38, p-p38, p-ERK, ERK, p-JNK, JNK, and NF-κB p65 antibodies were purchased from Cell Signaling Technology (Danvers, MA, USA). Anti-CD11c and YM1/Chitinase 3-like 3 (Chi3l3) antibodies were purchased from R&D Systems (Minneapolis, MN, USA). Anti-PHLPP1 antibody was purchased from Millipore (MA, USA). Anti-CYP2J2 antibody was purchased from Proteintech (ProteinTech Group, Chicago, IL, USA). Horseradish peroxidase-conjugated secondary antibodies were purchased from Thermo Fisher Scientific (Rockford, IL).

**Histology and immunohistochemistry**

Epididymal fat samples were fixed in 10% formalin, dehydrated, and embedded in paraffin. The 5μm thick sections were stained with hematoxylin and eosin to examine tissue morphology. Infiltration of macrophages in the epididymal adipose tissue were examined by immunohistochemistry. Briefly, 10μm serial sections were dewaxed and epitope retrieval was performed by immerging the slides into a citrate buffer (Boster, China) at 100°C for 20 min. After blocking with 5% goat serum blocking buffer for 1 h and staining overnight with antibodies against F4/80 (dilution 1:150), the sections were further incubated with peroxidase-conjugated secondary antibodies and DAB, then counterstained with hematoxylin. The histological characterizations, including adipocyte size and ATM crown-like structure were examined. Five random fields from each section were examined and Image-Pro Plus Version 6.0 was used to measure adipocyte average area. For quantification of crown-like structure (CLS) frequency in epididymal fat sections, CLSs were defined as necrotic-like adipocytes completely surrounded by nonadipocyte cells. At least 1,000 adipocytes per mouse were analyzed.

**Western blotting analysis**

Protein extracts from tissue and cultured cells were obtained using ice-cold RIPA lysis buffer (50mM Tris HCl, pH7.4, 150 mM NaCl, 1%NP-40, 0.5% sodium deoxycholate, 0.1% SDS) supplemented with protease and phosphatase inhibitors (Roche Applied Science) and centrifuged at 4˚C and 12000 g for 15 min. The protein concentration was measured using a standard Western blotting protocol as described previously (Imai *et al.,* 2008). Briefly, totally 40 μg protein was subjected to 10% SDS-PAGE gel for separation, and then transferred into PVDF membrane (Millipore, Bedford, USA). After blocking with 5％ non-fat milk for 2h, the membranes were incubated with a 1:1,000 dilution of primary antibodies overnight at 4℃. After washing by TBST, the membranes were incubated with a 1:5,000 dilution of horseradish peroxidase-conjugated secondary antibody for 1.5h at room temperature. Bands were visualized using ECL (Thermo Scientific Pierce) and quantified with Gel Pro Analyzer version 6.0 (Media Cybernetics, Bethesda, MD).

**Detection of cytokine and adipokine production**

Mice were anesthetized with pentobarbital (50 mg/kg), and cardiac puncture was performed. Blood was mixed with EDTA (1.5mg/ml), followed by centrifugation at 9000g for 10 min at 4°C for plasma collection. TNF-α, IL-6, MCP-1, adiponectin, leptin and resistin levels in serum were measured using ELISA kits according to the manufacturer's instructions (Boster Bio, Pleasanton, CA, USA).

**Muscle Blood Flow Measurement**

Muscle blood flow was measured with the fluorescent microsphere method as previously described (Kubis *et al.,* 2002). Briefly, A catheter was inserted in the carotid artery for injection of fluorescent microspheres, a second one was inserted in the femoral artery for reference blood sample withdrawal. After stabilization of hemodynamic parameters, 200 µl of yellow-green fluorescent microspheres (Triton, CA, USA) were injected into the carotid artery with an injection syringe over 10 s followed by 0.1 ml of saline. A reference blood sample was withdrawn from the femoral artery at a rate of 0.25 ml/min, into a pre-weighed heparinated syringe, starting 10 s before microsphere injection and lasting for a total of 60 s. The syringe containing the blood sample was weighed and the blood was digested with 250 µl of 16N KOH. Mice were sacrificed and skeletal muscles were weighed and digested in 4 ml of 4N KOH with 20% Tween 80. After 24 h, the digested tissues were filtered individually and processed for fluorescence quantification.

**Blood pressure measurement**

Systolic blood pressure in the conscious state was measured by the indirect tail cuff method with a blood pressure monitor (BP-2010A, Softron, Japan) according to the manufacturer’s instructions. At least 6 readings were obtained for each experiment, and a mean value was assigned to each individual mouse.

**Body composition measurement**

Body composition was determined using the Bruker’s minispec LF 50 Whole Body Composition Analyzer, with a 0.17-T magnetic field, using a 7.5-MHz frequency pulse. Briefly, different group of mice were inserted into the bore without anesthesia. The mass of fat and lean tissue were measured within 2 minutes.

**Citrate synthase activity measurement**

Citrate synthase-specific activity was measured in the presence of 0.2 mM DTNB, 0.1 mM Triton X-100, and acetyl CoA 0.1 mM, all diluted in 100 mM Tris-HCl. The reaction was initiated by the addition of 20 mM oxaloacetate. The reaction catalyzed by citrate synthase produces TNB (thionitrobenzoic acid) which can be determined spectrophotometrically at 412 nm.

**Measurement of ATP synthesis**

ATP was quantified in mitochondrial aliquots by an ATP bioluminescent assay kit (Sigma, St. Louis, MO, USA) according to the manufacturer’s instructions. ATP is consumed and light is emitted when firefly luciferase catalyzes the oxidation of D-luciferin. ATP levels were measured as nmoles/min/mg of mitochondrial protein.

**Mitochondrial isolation and assays of respiratory chain complexes**

Mitochondria were isolated by differential centrifugation, as previously described (Lanza & Nair, 2009). For all enzyme assays, mitochondria (500 microg/ml) were dissolved in different buffers (indicated under specific enzyme assay) as described previously (Sun *et al.,* 2006).
